# Procyanidin B2 alleviates oxidized low-density lipoprotein-induced cell injury, inflammation, monocyte chemotaxis, and oxidative stress by inhibiting the nuclear factor kappa-B pathway in human umbilical vein endothelial cells

**DOI:** 10.1186/s12872-024-03858-3

**Published:** 2024-04-29

**Authors:** Limei Yuan, Lihua Fan, Zhiqiang Zhang, Xing Huang, Qingle Liu, Zhiguo Zhang

**Affiliations:** Department of Cardiovascular, Henan University of Chinese Medicine, 63 Dongming Road, Henan province, Zhengzhou, 450063 China

**Keywords:** Procyanidin B2, ox-LDL, Monocyte, Inflammation, Oxidative stress, Endothelial cells

## Abstract

**Background:**

Oxidized low-density lipoprotein (ox-LDL) can initiate and affect almost all atherosclerotic events including endothelial dysfunction. In this text, the role and underlying molecular basis of procyanidin B2 (PCB2) with potential anti-oxidant and anti-inflammatory activities in ox-LDL-induced HUVEC injury were examined.

**Methods:**

HUVECs were treated with ox-LDL in the presence or absence of PCB2. Cell viability and apoptotic rate were examined by CCK-8 assay and flow cytometry, respectively. The mRNA and protein levels of genes were tested by RT-qPCR and western blot assays, respectively. Potential downstream targets and pathways of apple procyanidin oligomers were examined by bioinformatics analysis for the GSE9647 dataset. The effect of PCB2 on THP-1 cell migration was examined by recruitment assay. The effect of PCB2 on oxidative stress was assessed by reactive oxygen species (ROS) level, malondialdehyde (MDA) content, and mitochondrial membrane potential (MMP).

**Results:**

ox-LDL reduced cell viability, induced cell apoptosis, and facilitated the expression of oxidized low-density lipoprotein receptor 1 (LOX-1), C-C motif chemokine ligand 2 (MCP-1), vascular cell adhesion protein 1 (VCAM-1) in HUVECs. PCB2 alleviated ox-LDL-induced cell injury in HUVECs. Apple procyanidin oligomers triggered the differential expression of 592 genes in HUVECs (|log_2_fold-change| > 0.58 and adjusted p-value < 0.05). These dysregulated genes might be implicated in apoptosis, endothelial cell proliferation, inflammation, and monocyte chemotaxis. PCB2 inhibited C-X-C motif chemokine ligand 1/8 (CXCL1/8) expression and THP-1 cell recruitment in ox-LDL-stimulated HUVECs. PCB2 inhibited ox-LDL-induced oxidative stress and nuclear factor kappa-B (NF-κB) activation in HUVECs.

**Conclusion:**

PCB2 weakened ox-LDL-induced cell injury, inflammation, monocyte recruitment, and oxidative stress by inhibiting the NF-κB pathway in HUVECs.

**Supplementary Information:**

The online version contains supplementary material available at 10.1186/s12872-024-03858-3.

## Introduction

Atherosclerosis (AS), the formation of fibrofatty lesions in the artery wall, is the prominent pathophysiological process of multiple cardiovascular events such as stroke, heart attack, and myocardial infarction, while cardiovascular diseases are the principal contributors and causes of physical disability and deaths worldwide [1, 2]. The occurrence and development of AS can be induced and aggravated by multiple risk factors such as high-fat diet, senescence, obesity, and physical inactivity [1, 3].

The endothelium has been well-documented to be a crucial cellular monolayer in the maintenance of multi-organ health and homeostasis and to be a vital player in multiple biological processes such as regulation of vascular tone, substance exchange/transport and innate immunity, mechanotransduction, vascular injury repair, hemostasis, angiogenesis, and metabolism [4]. Endothelial dysfunction, implicated in multiple biological responses such as inflammation, endothelial cell injury and death, oxidative stress, and leukocyte adhesion/transmigration, is a common feature of multiple diseases including AS [4]. Many clinically used pharmacotherapies and drug candidates for atherosclerotic cardiovascular diseases and beyond can counter endothelial dysfunction [4]. The development and discovery of drugs targeting the endothelium and endothelial cell dysfunction might contribute to the clinical treatment of cardiovascular diseases including AS.

Oxidized low-density lipoprotein (ox-LDL) can accelerate AS progression by inducing endothelial damage and oxidative stress, expression of pro-inflammatory factors and adhesion molecules, and monocyte recruitment [5–7]. Oxidized low-density lipoprotein receptor-1 (LOX-1), a major receptor for ox-LDL in multiple cells including monocytes and endothelial cells, functions as a crucial player in the uptake of ox-LDL by cells [8, 9]. The internalization of ox-LDL in endothelial cells can activate apoptotic-related pathways and induce the expression of adhesion factors including intercellular adhesion molecule-1 (ICAM-1), vascular cell adhesion molecule-1 (VCAM-1), and chemokine (c-c motif) ligand 2 (MCP-1) [6, 9]. The resulting endothelial cell injury and increased adhesion molecules (e.g. ICAM-1, VACM-1, and MCP-1) can induce the recruitment of monocytes and initiation of AS [10, 11].

Procyanidins, a class of flavonoids that are widely existed in many plant sources such as fruits, vegetables, cereals, and tea, possess various potent pharmacological properties such as anti-oxidant, anti-inflammatory, antidiabetic, and anti-aging activities [12, 13]. Procyanidins have been reported to be beneficial for multiple disorders including cardiovascular and inflammatory diseases and diabetes [14]. Moreover, certain studies have demonstrated that procyanidins could alleviate AS progression in animal models [15–17]. Procyanidin B2 (PCB2), has been reported to be a potential protective agent in human umbilical vein endothelial cell (HUVEC) injury induced by some factors such as glycated low-density lipoproteins [18], advanced glycation-end products [19], and lipopolysaccharides (LPS) [20]. However, the roles and molecular mechanisms of PCB2 in AS development and ox-LDL-induced HUVEC cell injury have not been extensively explored.

In this text, the roles and downstream regulatory mechanisms of PCB2 underlying AS progression were examined in the ox-LDL-induced HUVEC cell injury model in vitro.

## Materials and methods

### Reagent, cell culture, and cell intervention

Ox-LDL was obtained from Yeasen Biotechnology Co., Ltd. (Shanghai, China). PCB2 was purchased from Selleck Co., Ltd. (Shanghai, China). HUVECs were purchased from American Type Culture Collection (ATCC, Manassas, VA, USA) and maintained in Ham’s F-12 K medium supplemented with 0.1 mg/mL Heparin, 0.03 mg/mL Endothelial Cell Growth Supplement, 10% fetal bovine serum (FBS), and 1% penicillin/streptomycin (cat. no. CM-0122, Procell Life Science&Technology Co., Ltd., Wuhan, China). For the ox-LDL-treated cell model, HUVECs were treated with 100 µg/ml of ox-LDL for 24 h. After 24 h of ox-LDL (100 µg/ml) treatment, HUVECs were cultured in the complete medium containing low (L, 0.5 µg/ml), medium (M, 1 µg/ml), or high (H, 2 µg/ml) concentration of PCB2 for another 12 h.

THP-1 human leukemia monocytic cell line, isolated from peripheral blood from an acute monocytic leukemia patient, is often used as a cell model to investigate the functions and activities of monocytes and macrophages along with related regulatory mechanisms [21]. THP-1 cells (Procell Life Science&Technology) were cultured in RPMI-1640 medium containing 10% FBS, 0.05 mM β-mercaptoethanol, and 1% penicillin/streptomycin (cat. no. CM-0233, Procell Life Science&Technology).

### Cell counting kit-8 (CCK-8) assay

Cell viability was analyzed by CCK-8 assay using the CCK-8 kit (Beyotime Biotechnology Co., Ltd., Shanghai, China) according to the protocols of the manufacturer. Briefly, cells were seeded into 96-well plates and treated with ox-LDL alone (100 µg/ml) for 24 h or along with different concentrations of PCB2 (0.5, 1, or 2 µg/ml) for an additional 12 h. At the indicated time points after treatment, CCK-8 solution (10 µl per well) was inoculated into 96-well plates and incubated for 1 h. Next, the absorbance was measured at 450 nm.

### Cell apoptotic rate detection

Cell apoptotic rate was examined using the Annexin V-FITC Apoptosis Detection Kit (Beyotime Biotechnology) referring to the manufacturer’s protocols. Briefly, HUVECs were stimulated with ox-LDL (100 µg/ml) for 24 h and then treated with different concentrations of PCB2 (0, 0.5, 1, or 2 µg/ml) for an additional 12 h. HUVECs were collected and resuspended in Annexin V-FITC binding solution. Next, HUVECs were incubated with Annexin V-FITC and propidium iodide staining solution for 15 min at 20–25˚C in a dark environment. Finally, the cell apoptotic rate was measured by flow cytometry. The cell percentage in the Q2 and Q3 regions denoted the apoptotic rate.

### Reverse transcription-quantitative PCR (RT-qPCR) assay

HUVECs were stimulated with DMSO or ox-LDL (100 µg/ml) for 24 h. After 24 h of ox-LDL (100 µg/ml) stimulation, HUVECs were treated with or without PCB2 (1 µg/ml) for an additional 12 h. Next, total RNA was isolated from HUVECs using the TRI Reagent Solution (Thermo Scientific, Waltham, MA, USA) following the manufacturer’s instructions. The synthesis of the cDNA first strand was performed using the above RNA template and RevertAid First Strand cDNA Synthesis Kit (Thermo Scientific) according to the manufacturer’s instructions. Quantitative PCR reactions were conducted using the SYBR Select Master Mix (Thermo Scientific) and specific primers for LOX-1, MCP-1, VCAM-1, ICAM-1, CXCL1, and CXCL8 on ABI QuantStudio6 Felx Real-Time System (Thermo Scientific). The relative expression levels of genes were measured using the 2^−ΔΔCt^ method. The primer sequences were shown in Table 1.

**Table 1:** The primer sequences for RT-qPCR

	Forward primer (5’-3’)	Reverse primer (5’-3’)
LOX-1	TTACTCTCCATGGTGGTGGTGCC	AGCTTCTTCTGCTTGTTGCC
MCP-1	CATAGCAGCCACCTTCATTCC	TCTCCTTGGCCACAATGGTC
VCAM-1	TGCCCATCTATGTCCCTTGC	GTCAACCCAGTGCTCCCTTT
ICAM-1	TTTGTTAGCCACCTCCCCAC	GCATATTCCCTGGGCACTCA
CXCL1	AGGCAGGGGAATGTATGTGC	AGCCCCTTTGTTCTAAGCCA
CXCL8	ATGACTTCCAAGCTGGCC	CAGACAGAGCTCTCTTCC
GAPDH	CCCTTCATTGACCTCAACTACATGG	AGTCTTCTGGGTGGCAGTGATGG

### Western blot assay

HUVECs were collected after 24 h of ox-LDL (100 µg/ml) stimulation alone or along with 12 h of PCB2 (1 µg/ml) treatment and then lysed using the RIPA lysis buffer (Beyotime Biotechnology) supplemented with protease inhibitor cocktail (Beyotime Biotechnology). After the quantitative analysis using the BCA Protein Assay Kit (Beyotime Biotechnology), protein (35 µg per lane) was separated by SDS-PAGE and transferred onto polyvinylidene fluoride membranes (Millipore, Bedford, MA, USA). Next, the membranes were sequentially incubated with 5% skimmed milk (1 h, room temperature), the primary antibody against LOX-1 (cat. no. 17958-1-AP, Proteintech, Wuhan, China), nuclear factor kappa-B (NF-κB) p65 (cat. no. 80979-1-RR, Proteintech, Rosemont, IL, USA), IKBα (cat. no. 10268-1-AP, Proteintech), IKBα (phospho S36) (cat. no. ab133462, Abcam, Cambridge, UK), Lamin B (cat. no. 12987-1-AP, Proteintech), GAPDH (cat. no. GB11002, Servicebio, Wuhan, China), or β-actin (cat. no. GB11001, Servicebio) and a secondary antibody conjugated with horseradish peroxidase (1 h, room temperature) (cat. no. G1213, Servicebio, Wuhan, China) were used. Finally, Pierce ECL Western Blotting Substrate (Thermo Scientific) was used to detect the protein signals.

### Enzyme linked immunosorbent assay (ELISA)

HUVECs were treated with ox-LDL (100 µg/ml) for 24 h. HUVECs after ox-LDL stimulation were incubated with or without PCB2 (1 µg/ml) for an additional 12 h. Next, cell supernatants were collected. MCP-1 (MultiSciences, Huangzhou, China), VCAM-1 (MultiSciences), ICAM-1 (MultiSciences), CXCL1 (Elabsciences, Wuhan, China) and CXCL8 (MultiSciences) secretion levels in the cell supernatants were measured using corresponding ELISA kits following the instructions of the manufacturer.

### Monocyte recruitment assay

HUVECs were treated with ox-LDL (100 µg/ml) for 24 h and stimulated with or without PCB2 (1 µg/ml) for another 12 h. Next, cell supernatants were collected and placed in the lower chambers of Transwell chambers (8-µm pore size, Costar Corning Inc., Corning, NY, USA). THP-1 cells were added to the upper chambers. Five hours later, THP-1 cells in the low chambers were stained with calcein, imaged, and counted.

### Bioinformatics analysis

Procyanidin oligomers belong to the procyanidins. To elucidate the downstream molecular targets underlying action of PCB2, we utilized the GSE9647 dataset that analyzed gene expression alterations in response to the stimulation of apple procyanidin oligomers in HUVECs. The GSE9647 dataset was downloaded from the Gene Expression Omnibus (GEO) database (https://www.ncbi.nlm.nih.gov/geo/query/acc.cgi?acc=GSE9647). Gene differential expression analysis in HUVECs treated with apple procyanidin oligomers and DMSO-treated controls based on the GSE9647 dataset was performed using the GEO2R software with the criterion of |log_2_fold-change| > 0.58 and adjusted p-value < 0.05. GO and KEGG enrichment analysis of these differentially expressed genes was performed using the KOBAS software [22].

### Oxidative stress index determination

HUVECs were stimulated with ox-LDL (100 µg/ml) for 24 h and then treated with different concentrations of PCB2 (0, 0.5, 1, or 2 µg/ml) for an additional 12 h.

Malondialdehyde (MDA) level was measured using a Lipid Peroxidation MDA Assay Kit (cat. no. S0131S, Beyotime Biotechnology) following the protocols of the manufacturer. MDA is an indicator of oxidative stress and a natural product of lipid oxidation. The lipid peroxidation MDA assay kit uses a color reaction of MDA and thiobarbituric acid (TBA) to yield the red MDA-TBA adduct.

The reactive oxygen species (ROS) level was examined by ROS Assay Kit (cat. no. S0033S, Beyotime Biotechnology) according to the instructions of the manufacturer. Oxidative stress can induce the accumulation of ROS. The ROS assay kit uses the fluorescent probe DCFH-DA to determine the ROS level. Briefly, DCFH-DA is a fluorescent probe that doesn’t produce fluorescence itself and can penetrate the cell membrane freely. Next, DCFH-DA is hydrolyzed into DCFH by the esterase in the cells. DCFH cannot pass through the cell membrane and is loaded into the cells. Non-fluorescent DCFH is oxidized into fluorescent DCF by ROS in the cells. Thus, the ROS level in the cells can be assessed by the fluorescent intensity of DCF.

Mitochondrial membrane potential (MMP) was determined through a mitochondrial membrane potential assay kit with tetraethylbenzimidazolylcarbocyanine iodide (JC-1) (cat. no. C2006, Beyotime Biotechnology) according to the manufacturer’s protocols. JC-1 is predominantly a monomer that produces green fluorescence when the MMP is low. At high MMP, JC-1 is gathered in the mitochondrial matrix to form JC-1 aggregates, which can yield red fluorescence. Thus, the relative percentage of red/green fluorescence is often used to examine the MMP.

### Statistical analysis

Data were analyzed using the GraphPad Prism software (Version 7, La Jolla, CA, USA) and the results were displayed as mean ± standard deviation. The differences between groups were examined by student’s *t*-test. The differences among groups were analyzed using one-way or two-way Analysis of Variance (ANOVA) along with the turkey test. The differences were defined to be statistically significant at *p*-value < 0.05.

## Results

### Ox-LDL induced cell injury and the expression of LOX-1 and adhesion molecules in HUVECs

**Fig. 1:**
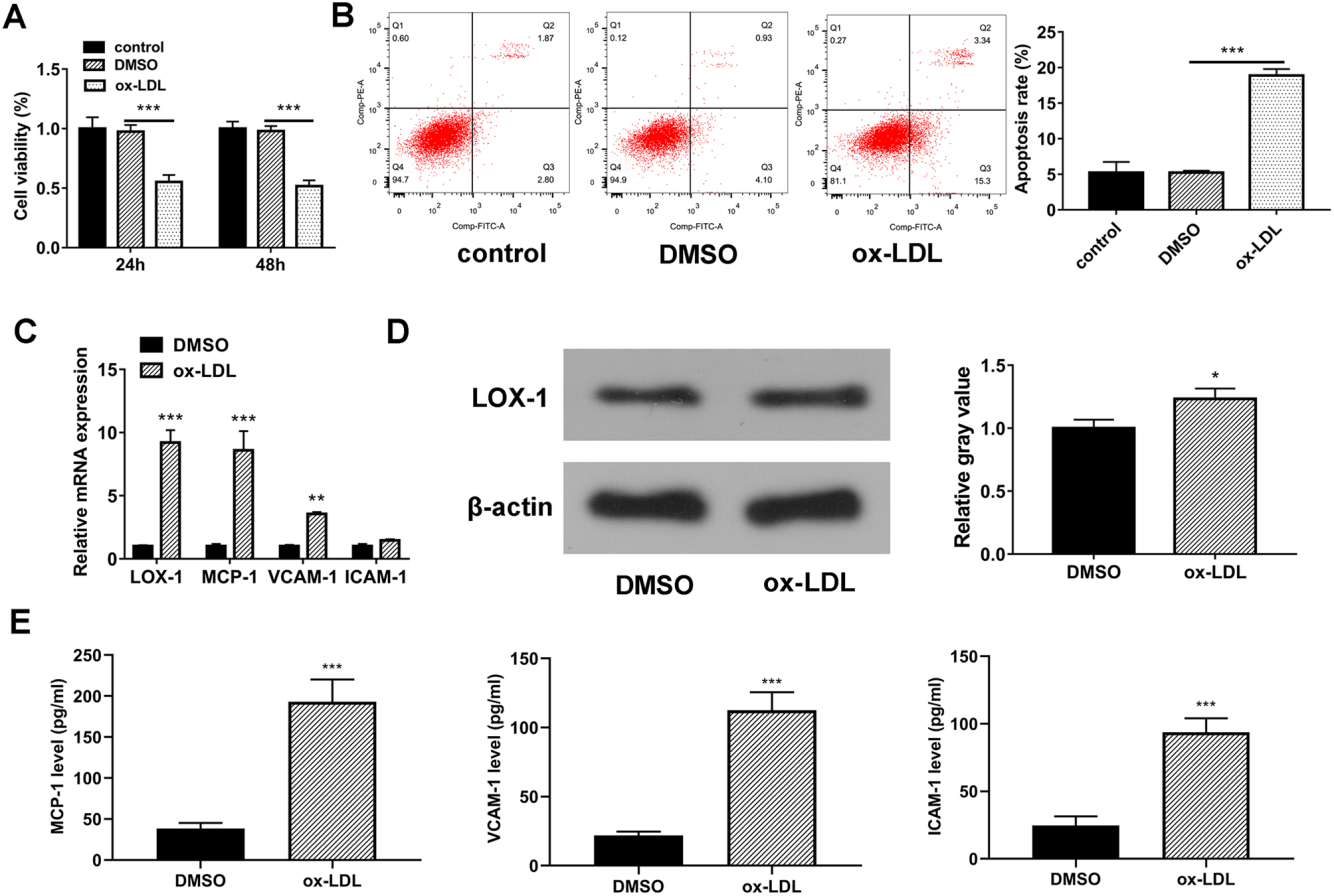
Ox-LDL induced cell injury and the expression of LOX-1 and adhesion molecules in HUVECs. (**A**) HUVECs were treated with DMSO or ox-LDL (100 µg/ml) for 24–48 h. Untreated cells functioned as the control group. Cell viability was examined by CCK-8 assay. (**B**-**D**) HUVECs were treated with DMSO or ox-LDL (100 µg/ml) for 24 h. DMSO-treated cells functioned as the control group. (**B**) Cell apoptotic pattern was tested by flow cytometry. (**C**) The mRNA levels of LOX-1, MCP-1, VCAM-1, and ICAM-1 were detected by RT-qPCR assay. (**D**) The protein level of LOX-1 was measured through western blot assay. (**E**) The secretion levels of MCP-1, VCAM-1 and ICAM-1 were gauged by ELISA. *n* = 3 independent biological replicates in A-D. *P < 0.05, **P < 0.01, ***P < 0.001

Prior studies have demonstrated that 100 µg/ml of ox-LDL can induce notable cell injury in HUVECs [23, 24]. Thus, 100 µg/ml of ox-LDL was used to induce HUVEC cell injury in this study. CCK-8 assay showed that cell viability was notably reduced in HUVECs after 24–48 h of ox-LDL (100 µg/ml) stimulation (Fig. 1A). Next, HUVECs were treated with ox-LDL (100 µg/ml) for 24 h given the similar cell viability inhibition at 24–48 h after ox-LDL stimulation (Fig. 1A). Flow cytometry analysis revealed that ox-LDL stimulation markedly increased cell apoptotic rate in HUVECs (Fig. 1B). These data suggested that ox-LDL exposure could induce HUVEC cell injury. The RT-qPCR assay also showed that ox-LDL treatment could trigger the notable increase of LOX-1, MCP-1, and VCAM-1 mRNA levels in HUVECs (Fig. 1C). Moreover, the western blot assay showed that the LOX-1 protein level was markedly elevated in HUVECs in response to ox-LDL stimulation (Fig. 1D). In addition, ox-LDL stimulation resulted in elevated secretion levels of MCP-1 and VCAM-1 in the culture supernatant of HUVECs (Fig. 1E). Although ox-LDL stimulation did not affect the mRNA expression of ICAM-1 in HUVECs, it caused a striking upregulation in ICAM-1 secretion level (Fig. 1E).

### PCB2 alleviated ox-LDL-induced HUVEC cell injury

**Fig. 2:**
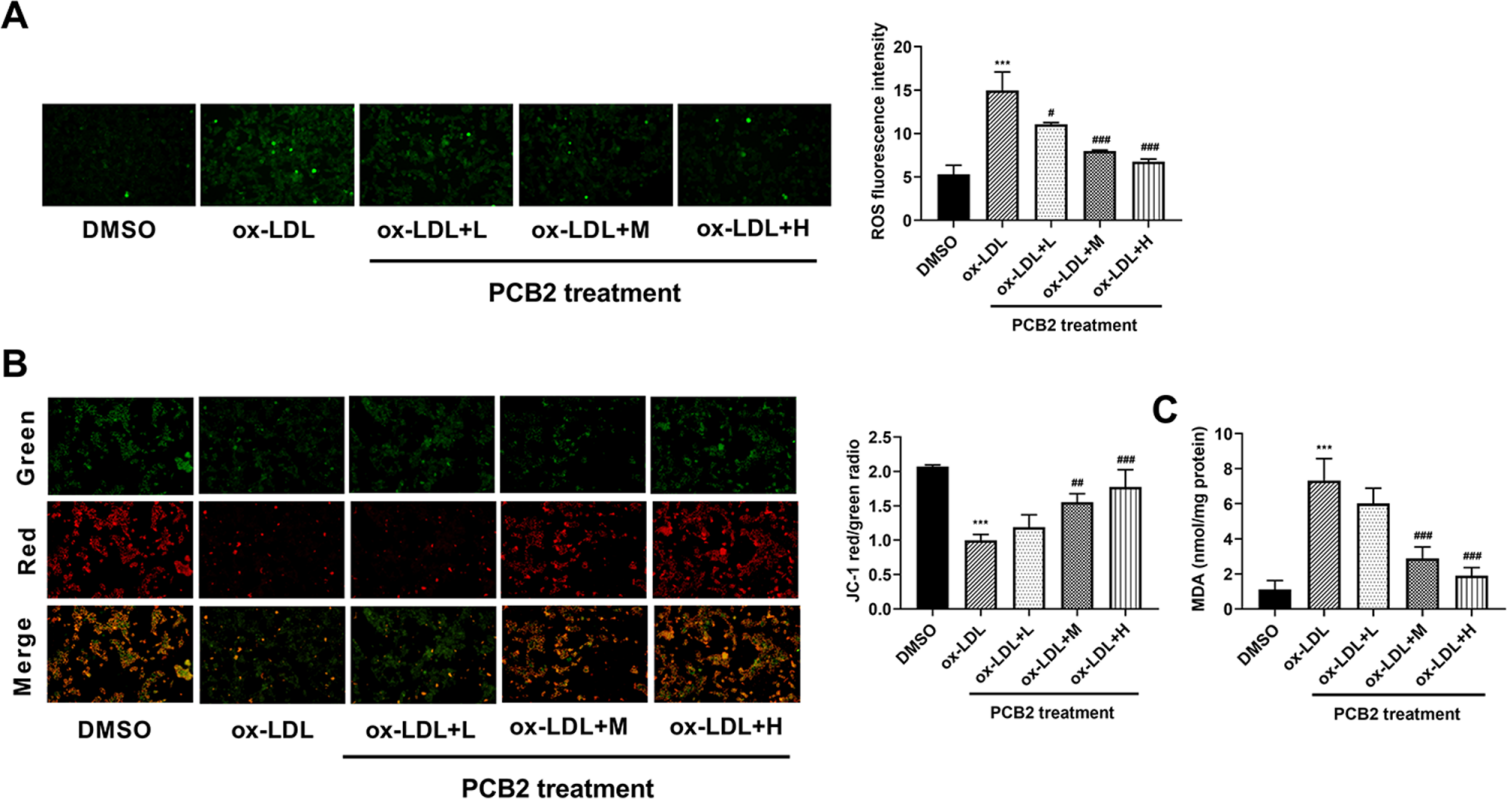
PCB2 alleviated ox-LDL-induced HUVEC cell injury. (**A** and **B**) HUVECs were stimulated with ox-LDL (100 µg/ml) for 24 h and then treated with different concentrations of PCB2 (0, 0.5, 1, or 2 µg/ml) for an additional 12 h. Untreated cells acted as the control group. Next, cell viability and apoptotic rate were examined by CCK-8 and flow cytometry, respectively. *n* = 3 independent biological replicates in A and B. ***P < 0.001, ^#^*P* < 0.05, ^##^*P* < 0.01, ^###^*P* < 0.001

Next, we further demonstrated that PCB2 relieved the inhibitory effect of ox-LDL on cell viability in a dose-dependent manner in ox-LDL-treated HUVECs (Fig. 2A). Moreover, PCB2 concentration-dependently inhibited ox-LDL-induced cell apoptosis in HUVECs (Fig. 2B). These data showed that PCB2 weakened ox-LDL-induced HUVEC cell injury in a concentration-dependent fashion. Given the similar protective effect of 1–2 µg/ml of PCB2 against ox-LDL-induced HUVEC cell injury, 1 µg/ml of PCB2 was used in the following experiments.

### PCB2 protected HUVECs from ox-LDL-induced oxidative stress

**Fig. 3:**
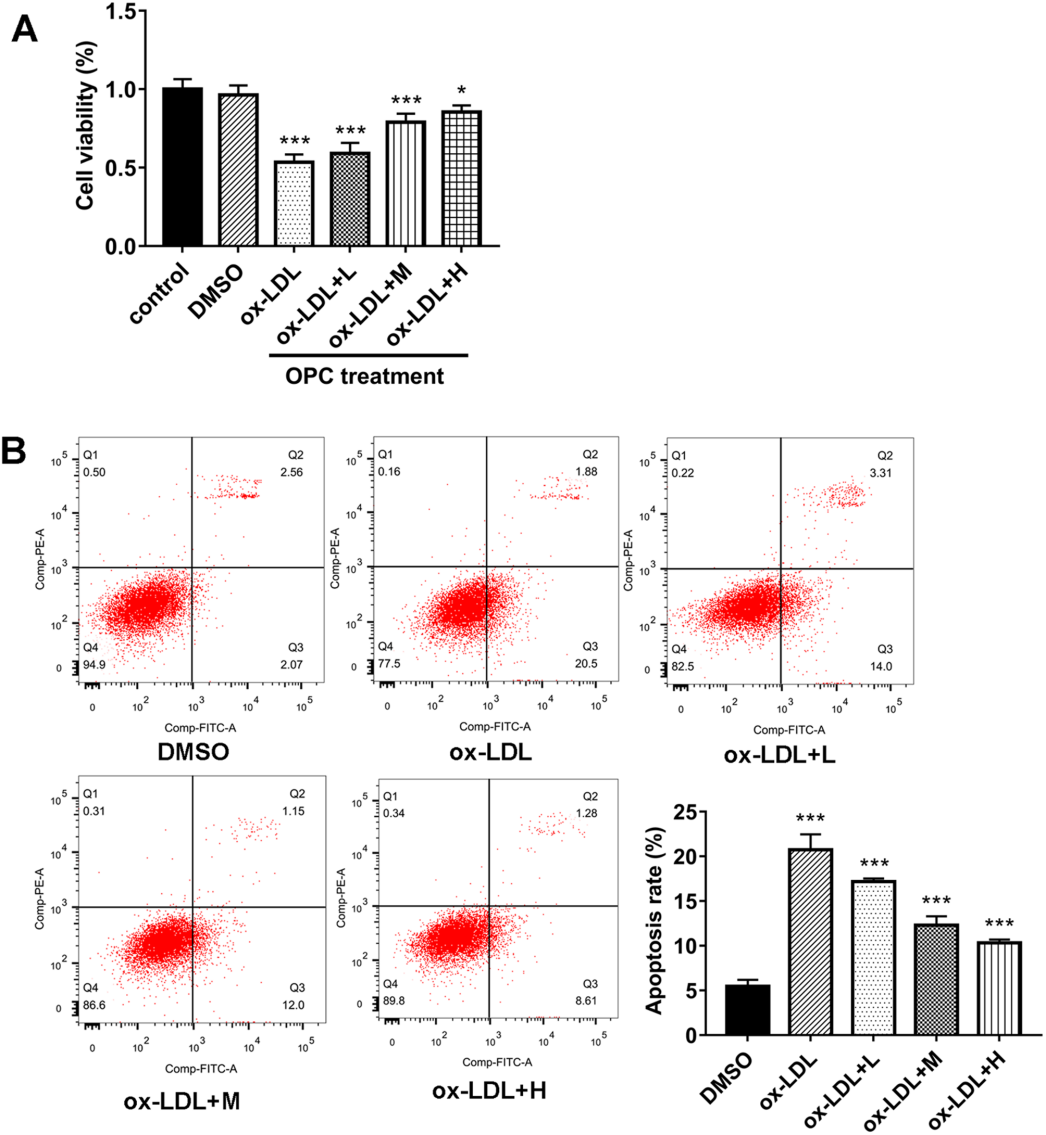
PCB2 protected HUVECs from ox-LDL-induced oxidative stress. (**A**-**C**) HUVECs were stimulated with ox-LDL (100 µg/ml) for 24 h and then treated with different concentrations of PCB2 (0, 0.5, 1, or 2 µg/ml) for an additional 12 h. Next, MDA, ROS, and MMP levels were measured by corresponding kits. *n* = 3 independent biological replicates in A-C. ***P < 0.001, ^#^*P* < 0.05, ^##^*P* < 0.01, ^###^*P* < 0.001

Given the close association of inflammation and oxidative stress in endothelial dysfunction [4, 25], we further explored the effect of PCB2 on oxidative stress in ox-LDL-stimulated HUVECs. Results showed that ROS level was markedly raised in HUVECs following ox-LDL stimulation and PCB2 suppressed ox-LDL-induced ROS production in a concentration-dependent fashion in HUVECs (Fig. 3A). Additionally, lower MMP was observed in ox-LDL-treated HUVECs than in DMSO-treated cells (Fig. 3B). And, the introduction of different concentrations of PCB2 led to a dose-dependent increase of MMP in ox-LDL-treated HUVECs (Fig. 3B). ox-LDL exposure triggered the notable elevation of MDA level and PCB2 concentration-dependently curbed the increase of MDA content provoked by ox-LDL stimulation in HUVECs (Fig. 3C). In summary, these outcomes showed that PCB2 weakened ox-LDL-induced oxidative stress in HUVECs.

### Identification of potential downstream targets and signaling pathways of PCB2

**Fig. 4:**
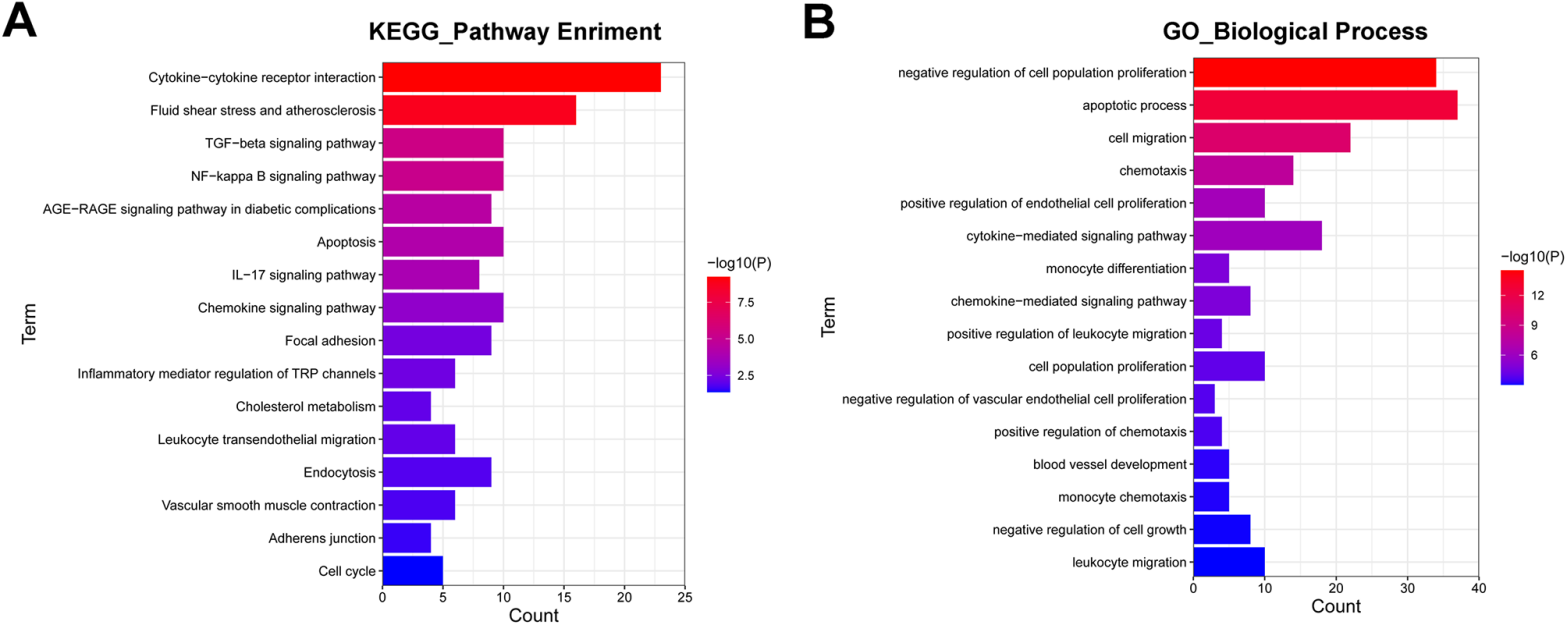
Identification of potential downstream targets and signaling pathways of PCB2. (**A** and **B**) The 16 representative KEGG pathways and GO biological processes that were significantly enriched by dysregulated genes after the stimulation of apple procyanidin oligomers in HUVECs

To have a deep insight into the mechanisms of action of PCB2 in ox-LDL-stimulated HUVECs, the GEO dataset GSE9647, which investigated gene expression alterations in response to the stimulation of apple procyanidin oligomers in HUVECs, was downloaded from the GEO database. Differential expression analysis for the GSE9647 dataset using the GEO2R software revealed that 261 genes were markedly down-regulated (log_2_fold-change < -0.58, adjusted p-value < 0.05) and 331 genes were notably up-regulated (log_2_fold-change > 0.58, adjusted p-value < 0.05) in HUVECs treated with apple procyanidin oligomers compared to the DMSO-treated group (Supplementary Table 1). Among the differentially expressed genes, CXCL1 and CXCL8 were markedly down-regulated in HUVECs following the treatment of apple procyanidin oligomers (Supplementary Table 1). KEGG enrichment analysis showed that these dysregulated genes were involved in the regulation of cytokine-cytokine receptor interaction, apoptosis, chemokine signaling pathway, and leukocyte transendothelial migration (Fig. 4A, Supplementary Table 2). GO biological process enrichment analysis disclosed that these differentially expressed genes after the stimulation of apple procyanidin oligomers might play vital roles in biological processes related to vascular endothelial cell proliferation, apoptotic process, cell migration, chemotaxis, cytokine/chemokine-mediated signaling pathway, and monocyte chemotaxis (Fig. 4B, Supplementary Table 3).

### PCB2 inhibited the expression and secretion of CXCL1 and CXCL8 and THP-1 cell recruitment in ox-LDL-treated HUVECs

**Fig. 5:**
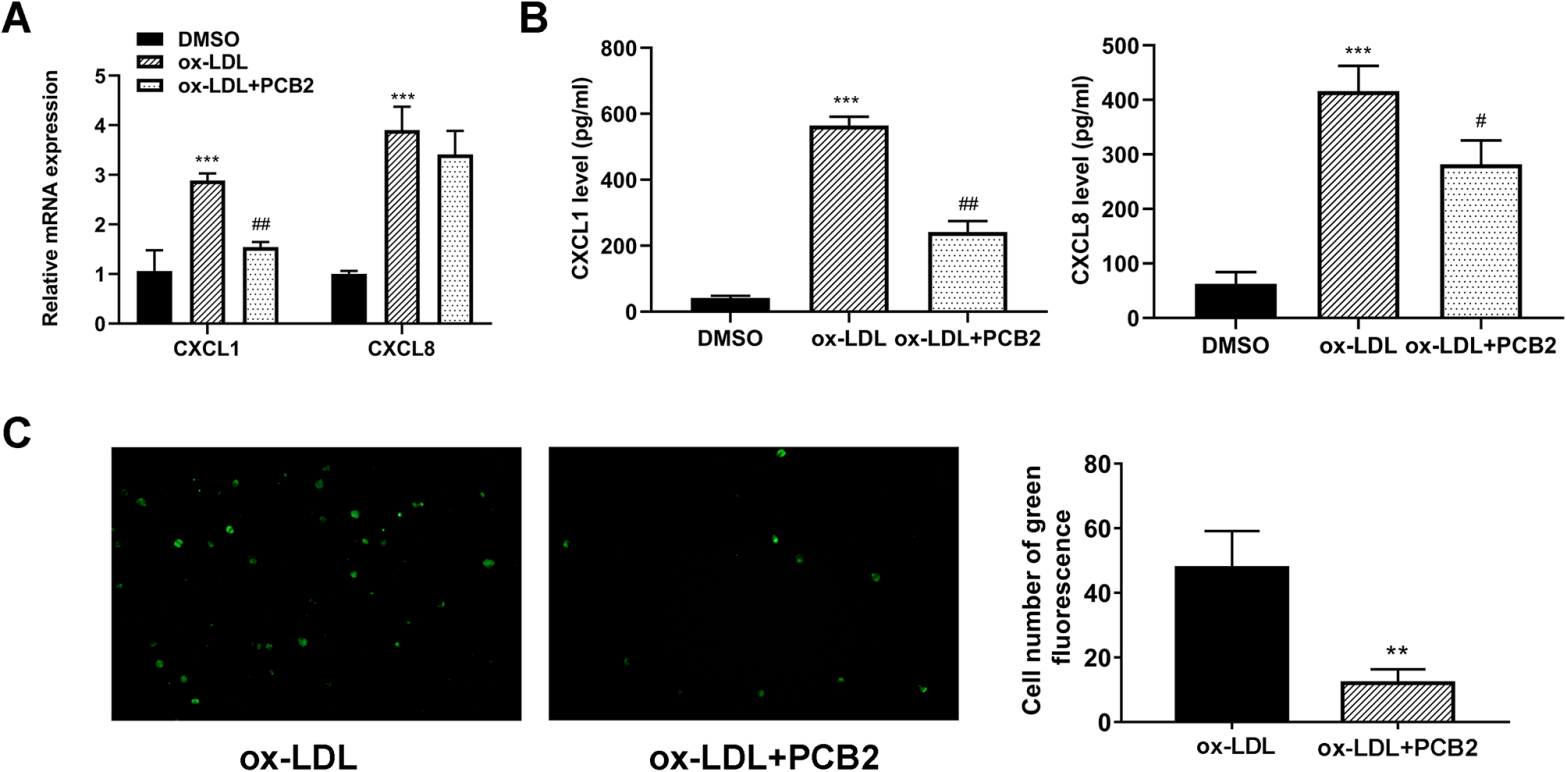
PCB2 inhibited the expression of CXCL1 and CXCL8 and recruitment of THP-1 cells in ox-LDL-treated HUVECs. (**A** and **B**) HUVECs were stimulated with ox-LDL (100 µg/ml) or DMSO vehicle for 24 h and treated with or without PCB2 (1 µg/ml) for an additional 12 h. (**A**) CXCL1 and CXCL8 mRNA levels were measured by RT-qPCR assay. (**B**) CXCL1 and CXCL8 secretion levels were tested by ELISA assay. (**C**) HUVECs were treated with ox-LDL (100 µg/ml) for 24 h alone or along with PCB2 (1 µg/ml) for another 12 h. Then, cell supernatants were collected and added to the low chambers. THP-1 cells were added to the upper chambers. Five hours later, THP-1 cells in the low chambers were stained, imaged, and counted. *n* = 3 independent biological replicates in A-C. *P < 0.05, **P < 0.01

Based on the above-mentioned enrichment analysis, we supposed that PCB2 might participate in the regulation of cytokine-mediated signaling pathways and related genes such as CXCL1 and CXCL8. Subsequent RT-qPCR assay demonstrated that ox-LDL treatment resulted in enhanced mRNA expression of CXCL1 and CXCL8; PCB2 (1 µg/ml) stimulation led to a notable reduction of CXCL1 mRNA level in ox-LDL-treated HUVECs (Fig. 5A). ELISA assay disclosed that ox-LDL treatment elevated the secretion levels of CXCL1 and CXCL8 in HUVECs; PCB2 suppressed CXCL1 and CXCL8 secretion levels in HUVECs stimulated with ox-LDL (Fig. 5B). Moreover, PCB2 hindered THP-1 cell recruitment in ox-LDL-treated HUVECs (Fig. 5C).

### PCB2 inhibited ox-LDL-induced NF-κB activation in HUVECs

**Fig. 6:**
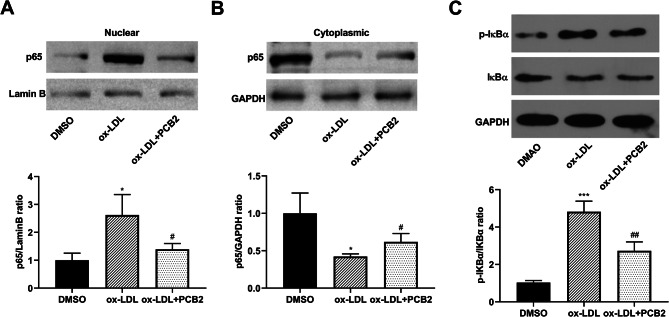
PCB2 inhibited ox-LDL-induced NF-κB activation in HUVECs. (**A**-**C**) HUVECs were stimulated with ox-LDL (100 µg/ml) for 24 h and treated with or without PCB2 (1 µg/ml) for an additional 12 h. Next, the protein levels of nuclear p65, cytoplasmic p65, IKBα, and p-IKBα were measured by western blot assay. *n* = 3 independent biological replicates in A-C. *P < 0.05, ***P < 0.001, ^#^*P* < 0.05, ^##^*P* < 0.01

Moreover, the western blot assay demonstrated that ox-LDL treatment led to a notable increase in nuclear p65 level, a marked reduction in cytoplasmic p65 level, as well as a strong augmentation in the p-IKBα/IKBα ratio in HUVECs (Fig. 6A and C), indicating that ox-LDL exposure could induce the activation of NF-κB signaling pathway in HUVECs. Additionally, the nuclear p65 level and p-IKBα/IKBα ratio were markedly reduced and the cytoplasmic p65 level was enhanced in ox-LDL-treated HUVECs following PCB2 stimulation (Fig. 6A and C). Collectively, PCB2 inhibited the activation of the NF-κB pathway in ox-LDL-treated HUVECs.

## Discussion

In this study, we demonstrated that PCB2 could attenuate ox-LDL-induced cell injury in HUVECs by repressing cell apoptosis and oxidative stress and suppress monocyte cell recruitment to ox-LDL-treated HUVECs. The protective function of PCB2 has recently been proved in attenuating AS progression by using related animal models [15–17]. Although PCB2 has been established as a potential protective agent in glycated low-density lipoproteins-induced HUVECs [18, 26], the current study for the first time unveiled the alleviating effect of PCB2 on apoptosis and oxidative stress of HUVECs induced by ox-LDL. More importantly, we first demonstrated that PCB2 is able to impede ox-LDL-induced monocyte cell recruitment to HUVECs. Mechanistically, the NF-κB signaling pathway is involved in the protective effect of PCB2 in ox-LDL-induced HUVECs. These findings provide novel evidence for the development of PCB2-based therapies in AS in the future.

To further investigate the biological responses after PCB2 stimulation in HUVECs, differential expression analysis for the GEO dataset GSE9647 was performed to examine the gene expression alterations in HUVECs following the treatment of apple procyanidin oligomers. Differential expression analysis for the GSE9647 dataset suggested that 592 genes, including CXCL1 and CXCL8, were differentially expressed (|log_2_ fold-change| > 0.58, adjusted p-value < 0.05) in HUVECs after the stimulation of apple procyanidin oligomers. KEGG and GO biological process enrichment analysis showed that these dysregulated genes mainly participated in the regulation of pathways or biological processes related to focal adhesion, inflammation, cytokine-related pathways, monocyte chemotaxis, and NF-κB signaling pathway.

It has been reported that inflammation and oxidative stress were closely linked and play vital roles in the development of cardiovascular diseases including AS [27, 28]. Moreover, inflammation and oxidative stress are implicated in endothelial dysfunction [4, 25]. NF-κB pathway also has been found to be closely linked with inflammation, oxidative stress, and endothelial dysfunction in AS [7, 29]. For instance, Chen et al. demonstrated that galectin-3 (Gal-3) potentiated ox-LDL-induced HUVEC cell injury and facilitated the expression of pro-inflammatory factors (interleukin [IL]-6, IL-8, IL-1β, CXCL-1, and CCL-2) and adhesion molecules (VCAM-1 and ICAM-1) via activating the NF-κB signaling pathway in ox-LDL-treated HUVECs [30]. Additionally, previous studies have suggested that procyanidins could alleviate AS by reducing oxidative stress [15, 17]. Thus, we further investigated the effect of PCB2 on CXCL1/CXCL8 expression, THP-1 monocyte recruitment, oxidative stress and the NF-κB signaling pathway in ox-LDL-treated HUVECs.

Our results suggested that PCB2 could inhibit ox-LDL-induced cell injury, pro-inflammatory cytokine secretion, THP-1 monocyte cell recruitment, oxidative stress, and NF-κB activation in HUVECs. Similar to our results, previous studies also suggested that PCB2 could increase cell viability, curb cell apoptosis and ROS production, improve MMP, inhibit pro-inflammatory cytokine secretion and the NF-κB signaling pathway in LPS-treated HUVECs [20, 31]. Also, prior studies suggested that procyanidins were involved in monocyte-endothelial adhesion [32, 33]. For instance, Lee et al. demonstrated that the extracts of procyanidin-rich Yak-Kong seed coat suppressed the adhesion of THP-1 cells to LPS-treated HUVECs [33]. Also, a recent study disclosed that PCB2 ameliorated endothelial dysfunction induced by soluble fms-like tyrosine kinase-1, reduced VCAM-1 expression in HUVECs, and inhibited leukocyte adhesion to HUVECs [34]. Our results demonstrated that PCB2 inhibited the expression and secretion of CXCL1 and CXCL8 in ox-LDL-treated HUVECs. Thus, we proposed that CXCL1 and CXCL8 might be two effectors of the protective role of PCB2 in atherosclerosis progression. The NF-κB pathway, a multi-functional pro-inflammatory signaling pathway, is strongly related to pro-inflammatory cytokine secretion, oxidative stress and cell apoptosis [35–37]. Based on the suppressive effect of PCB2 on ox-LDL-induced NF-κB activation, we concluded that the NF-κB signaling pathway is involved in the protective effect of PCB2 in ox-LDL-induced HUVECs. Similarly, Song et al. reported that PCB2 exerts a protective effect in LPS-induced HUVECs by the NF-κB signaling [31]. Previous reports show that several molecules, such as prohibitin and protein L-isoaspartyl methyltransferase, are responsible for the protective effect of PCB2 in endothelial cell injury induced by glycated low-density lipoproteins [18, 38]. More investigations will be done to elucidate whether these molecules are functional effectors of PCB2 in attenuating ox-LDL-induced HUVEC injury and AS progression. AS occurs in the artery wall. It is closer to clinical practice using arterial endothelial cells to study this disease pathogenesis. Our study using HUVECs is limited to uncover the function of PCB2 in ox-LDL-induced endothelial dysfunction in AS.

Taken together, our data showed that PCB2 weakened ox-LDL-induced HUVEC cell injury, reduced ox-LDL-induced endothelial cell inflammation and oxidative stress, and hindered ox-LDL-induced THP-1 cell adhesion to HUVECs, suggesting the potential endothelium-protective effect of PCB2 on ox-LDL-induced endothelial dysfunction and AS. The NF-κB signaling pathway is involved in the protective effect of PCB2 in ox-LDL-induced HUVECs. These data might contribute to the better understanding of the PCB2-mediated protective effect against ox-LDL-induced endothelial dysfunction and AS. Given the close association of endothelial dysfunction and the development of cardiovascular diseases including AS, PCB2 might have a potential therapeutic value for cardiovascular diseases. In the subsequent experiments, we intend to further investigate the effect of PCB2 on AS progression in various animal experiments.

### Electronic supplementary material

Below is the link to the electronic supplementary material.

Supplementary Material 1

Supplementary Material 2

Supplementary Material 3

## Data Availability

The data and materials displayed in this manuscript are available from the corresponding author upon reasonable request.

## Abbreviations

NF-κB: Nuclear factor kappa-B
ox-LDL: Oxidized low-density lipoprotein
PCB2: Procyanidin B2
ROS: Reactive oxygen species
MDA: Malondialdehyde
MMP: Mitochondrial membrane potential
LOX-1: Oxidized low-density lipoprotein receptor 1
MCP-1: C-C motif chemokine ligand 2
VCAM-1: Vascular cell adhesion protein 1
ICAM-1: Intercellular adhesion molecule-1
CXCL1: C-X-C motif chemokine ligand 1
CXCL8: C-X-C motif chemokine ligand 8
NF-κB: Nuclear factor kappa-B
AS: Atherosclerosis
HUVECs: Human umbilical vein endothelial cells
LPS: Lipopolysaccharides
ATCC: American Type Culture Collection
FBS: Fetal bovine serum
CCK-8: Cell Counting Kit-8
TBA: Thiobarbituric acid
RT-qPCR: Reverse transcription-quantitative PCR
SOD: Superoxide dismutase
Gal-3: Galectin-3
