# Supplementary material for: Procyanidin B2 alleviates oxidized low-density lipoprotein-induced cell injury, inflammation, monocyte chemotaxis, and oxidative stress by inhibiting the nuclear factor kappa-B pathway in human umbilical vein endothelial cells
Source: BMC Cardiovasc Disord. 2024 Apr 29;24:231. doi: 10.1186/s12872-024-03858-3 (PMC11057093; doi:10.1186/s12872-024-03858-3)

Figure 1D

LOX-1（repetition 1） LOX-1（repetition 2） LOX-1（repetition 3）


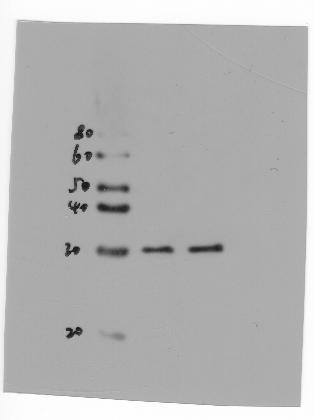

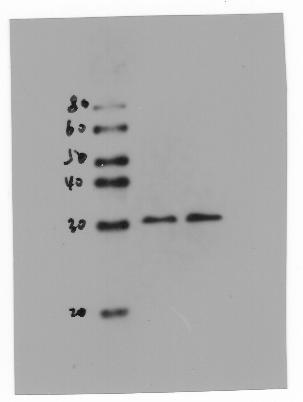

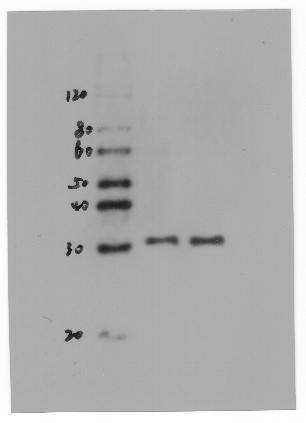


β-actin（repetition1） β-actin（repetition 2） β-actin（repetition 3）


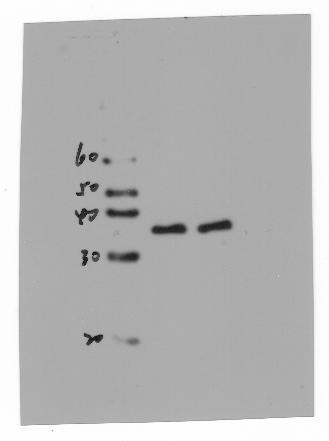

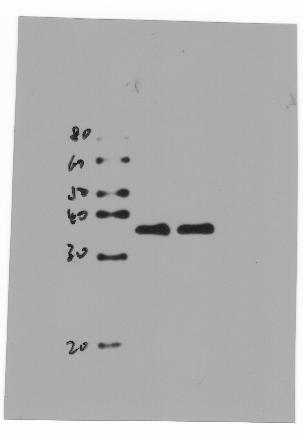

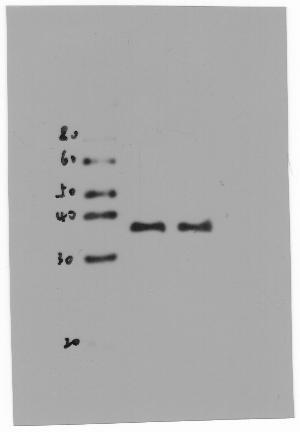


Figure 6

A

Nuclear P65 (repetition-1-2) Nuclear P65 (repetition-3)


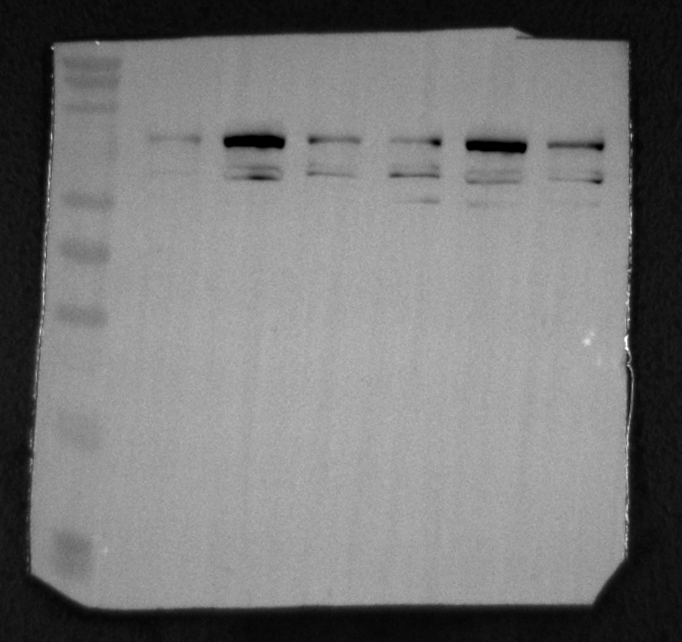

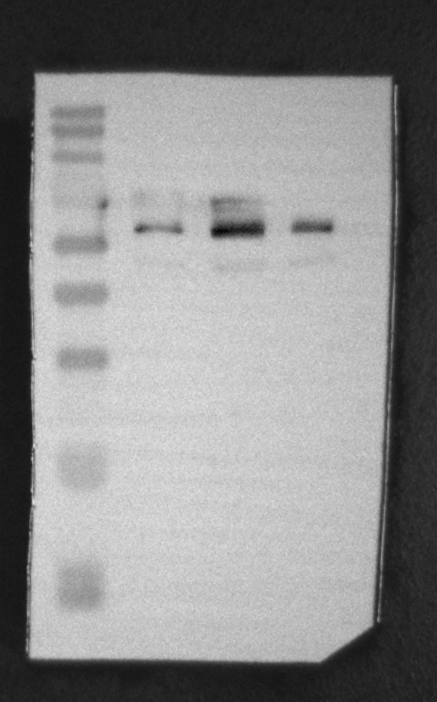


LaminB (repetition-1-2-3)


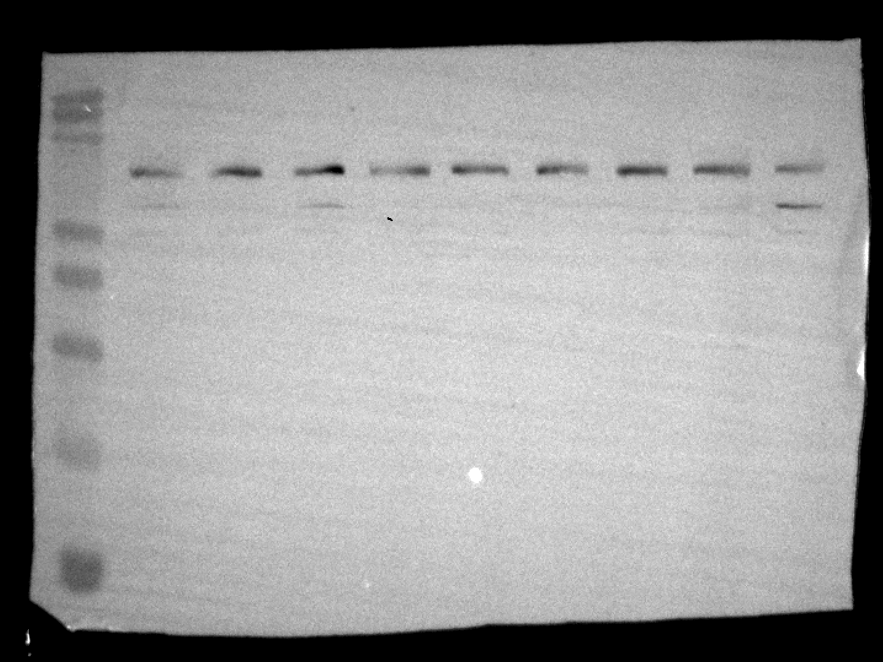


Figure 6

B

Cytoplasmic P65(repetition-1-2-3)


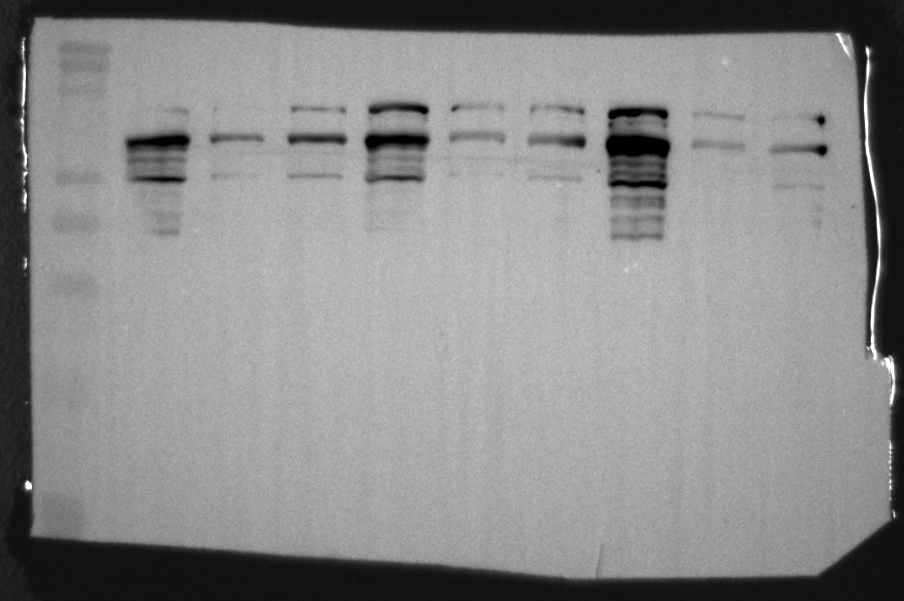


GAPDH(repetition-1-2-3)


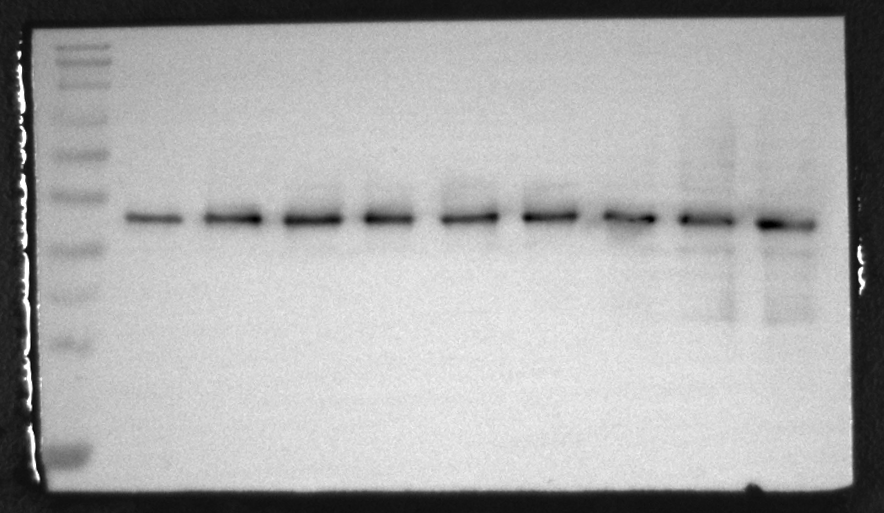


Figure 6

C

IκBα (repetition-1) IκBα(repetition-2) IκBα (repetition-3)


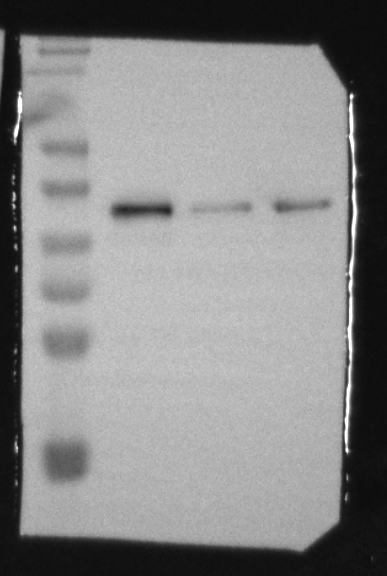

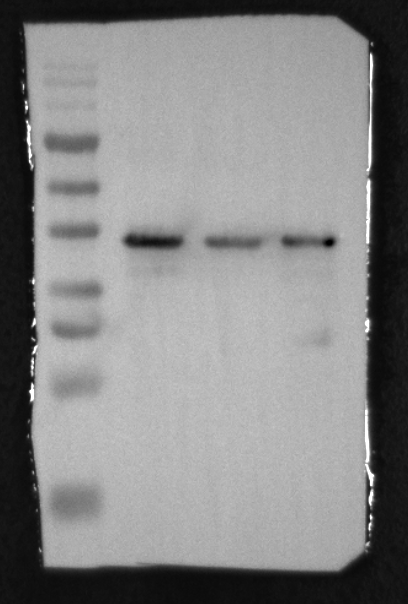

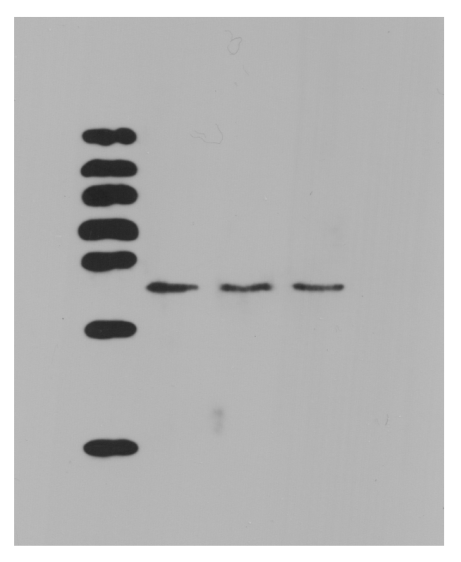


p-IκBα (repetition-1-2) p-IκBα (repetition-3)


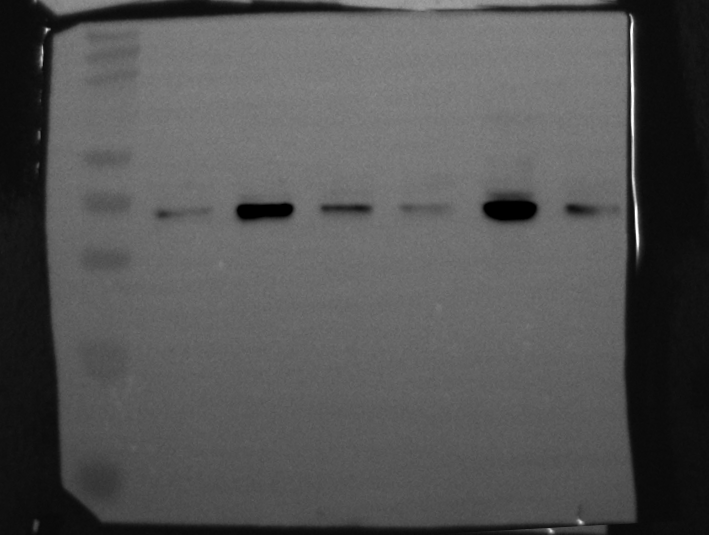

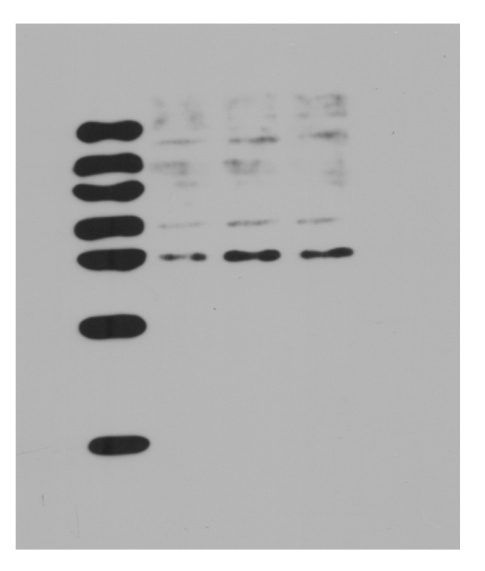


GAPDH（repetition-1） GAPDH（repetition-2） GAPDH (repetition-3)


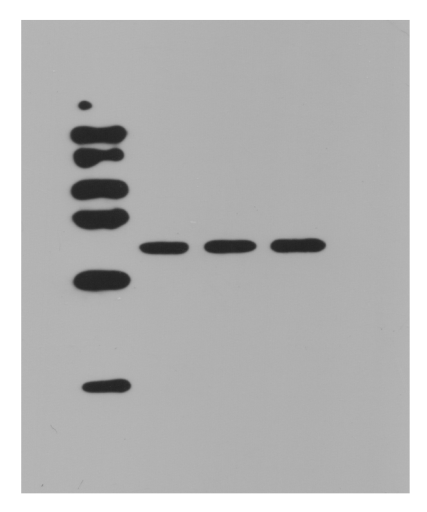

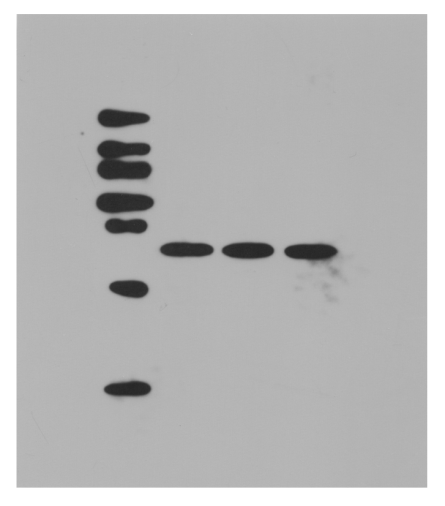

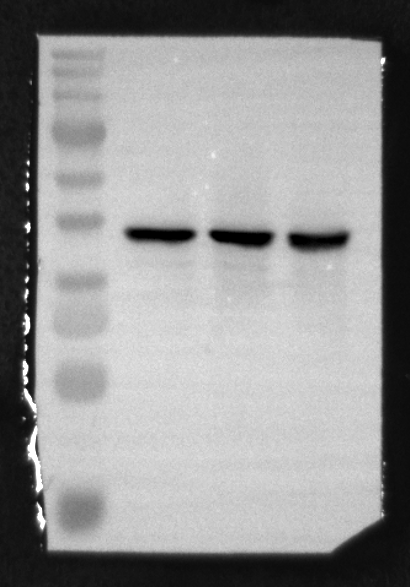

Supplement: Supplementary file 2 — Supplementary Material 2 [file 12872_2024_3858_MOESM2_ESM.docx]
