# Supplementary material for: Procyanidin B2 alleviates oxidized low-density lipoprotein-induced cell injury, inflammation, monocyte chemotaxis, and oxidative stress by inhibiting the nuclear factor kappa-B pathway in human umbilical vein endothelial cells
Source: BMC Cardiovasc Disord. 2024 Apr 29;24:231. doi: 10.1186/s12872-024-03858-3 (PMC11057093; doi:10.1186/s12872-024-03858-3)
Supplement: Supplementary file 3 — Supplementary Material 3 [file 12872_2024_3858_MOESM3_ESM.docx]

**Supplementary Table 1.** Differentially expressed genes after the stimulation of apple procyanidin oligomers in HUVECs.

**Supplementary Table 2.** KEGG enrichment analysis for differentially expressed genes after the stimulation of apple procyanidin oligomers in HUVECs.

**Supplementary Table 3.** GO biological process enrichment analysis for differentially expressed genes after the stimulation of apple procyanidin oligomers in HUVECs.
